# Retraction: Effect of rumex dentatus on gastrointestinal protection and toxicology in rodents via investigating H+/K+-ATPase, calcium channels, and PDE mediated signaling

**DOI:** 10.3389/fphar.2026.1858433

**Published:** 2026-05-07

**Authors:** Frontiers Editorial Office

The Journal retracts the 16 August 2022 article cited above.

Following publication, concerns were raised regarding the integrity of the images in the published figures. Image duplication concerns were identified between Figures 4F,I, as well as in Figures 7, 8.

The authors failed to provide a satisfactory explanation during the investigation, which was conducted in accordance with Frontiers’ policies. As a result, the data and conclusions of the article have been deemed unreliable and the article has been retracted.

This retraction was approved by the Chief Editors of Pharmacology and the Chief Executive Editor of Frontiers. The authors do not agree to this retraction.

